# Who seeks sex therapy? Sexual dysfunction prevalence and correlates, and help-seeking among clinical and community samples

**DOI:** 10.1371/journal.pone.0282618

**Published:** 2023-03-06

**Authors:** David Lafortune, Marianne Girard, Éliane Dussault, Mathieu Philibert, Martine Hébert, Marie-Aude Boislard, Mathieu Goyette, Natacha Godbout

**Affiliations:** Department of Sexology, Université du Québec à Montréal, Québec, Canada; University of Palermo: Universita degli Studi di Palermo, ITALY

## Abstract

Sexual dysfunctions (SD; e.g., female sexual interest/arousal disorder, erectile disorder, female orgasmic disorder, delayed ejaculation, genito-pelvic pain/penetration disorder, etc.) affect up to a third of individuals, impairing sexuality, intimate relationships, and mental health. This study aimed to compare the prevalence of SDs and their sexual, relational, and psychological correlates between a sample of adults consulting in sex therapy (*n* = 963) and a community-based sample (*n* = 1,891), as well as examine barriers to sexual health services for SD and the characteristics of individuals seeking such services. Participants completed an online survey. Analyses showed that participants in the clinical sample reported lower levels of sexual functioning and sexual satisfaction and higher levels of psychological distress than participants in the community-based sample. Moreover, higher SD rates were related to lower relational satisfaction and higher psychological distress in the community sample, and to lower sexual satisfaction in both samples. Among participants in the community sample who sought professional services for SD, 39.6% reported that they were unable to access services, and 58.7% reported at least one barrier to receiving help. This study provides important data regarding the prevalence of SD and the link between SD and psychosexual health in clinical and nonclinical samples, as well as barriers to treatment access.

## Introduction

### Prevalence of sexual dysfunctions

Sexual health is fundamental to well-being [1, 2]. However, sexual well-being has repeatedly been overshadowed in international public health agendas by other concerns like reproductive health and sexually transmitted infections [3, 4]. Yet, sexual dysfunctions (SD) are prevalent in the general population—impacting up to a third of adults regardless of age and gender [2, 5–10]. The fifth edition of the Diagnostic and Statistical Manual of Mental Disorders (DSM-5) [11] defines SD (e.g., male hypoactive sexual desire, erectile, or genito-pelvic pain/penetration disorders, etc.) as a significant impairment of sexual response and pleasure or as pain during intercourse, causing persistent (≥ 6 months) and clinically significant distress.

In their systematic review, Lewis and colleagues [7] reported that, among women, 17 to 55% meet the criteria for sexual interest/arousal disorder, 16 to 25% for orgasmic disorder, and 14 to 27% for genito-pelvic or penetration-related pain. Among men, 8 to 18% meet the criteria for hypoactive sexual desire disorder, 10 to 40% for erectile disorder, 8 to 30% for premature ejaculation, 1 to 10% for delayed ejaculation, and 1 to 6% for genito-pelvic or penetration-related pain [7]. Variations in SD estimates across studies are mostly due to differences in SD screening criteria and sampling methods and composition (e.g., clinical versus nonclinical samples) [7, 8, 12]. Regarding screening criteria, most SD studies only assess impairment criteria (*symptom-level*; e.g., low desire, erectile difficulties, ejaculation or orgasm latency) rather than examine all DSM-5 diagnostic criteria (*disorder-level*, i.e., minimum duration of 6 months and the presence of personal distress) [11]. Measuring SD this way is problematic from an intervention standpoint, as many individuals report impaired sexual function without experiencing distress [13, 14]. For instance, in a random population-based sample (*n* = 1,346), Hendrickx and colleagues [15] have found that, while 44% of women and 35% of men reported moderate to severe sexual difficulties (symptom-level), these percentages decreased to 19% and 15% respectively, when the DSM-5’s impairment duration and distress criteria were considered (disorder-level). Similarly, the Britain’s third *National Survey of Sexual Attitudes and Lifestyles* (*n* = 15,162) [16] revealed that more than 40% of men and 50% of women report at least one sexual problem, but that only about 10% also feel distressed regarding their sex lives. Furthermore, many contextual factors (e.g., postpartum period, professional stressors, romantic breakup) can temporarily impact sexual functioning [17, 18] without reaching clinical thresholds.

Recent studies on sexual functioning among individuals seeking clinical services (e.g., sex therapy, gynecology, family medicine) are scarce [19–23]. Studies that have compared individuals consulting in sex therapy to those of the general population have found that the former report lower levels of sexual functioning than the latter [24–27]. Yet, additional comparative research that uses disorder-level definitions of SD is needed to better understand the experiences of individuals consulting in clinical settings and guide the development of tailored interventions.

### Sexual, relational, and psychological correlates of sexual dysfunctions

SD impairs sexual and intimate relationships, as well as mental health [14]. Improving our understanding of the interactions between sexual functioning and relationship satisfaction is of great importance, given that about a third of men and women with low sexual functioning report being unsatisfied with their relationships [16]. SD has also been found to correlate negatively with sexual satisfaction [28–33]. Moreover, strong bidirectional associations between low sexual functioning and psychological distress were also found [16, 34]. However, findings regarding relationship satisfaction have been inconsistent, with some studies showing dyadic adjustment (i.e., relationship satisfaction) to be a key correlate of overall sexual functioning [9, 35–38], while other studies suggest that it is only weakly associated or unrelated with SD [20, 39]. These incongruencies highlight the need to further explore the links between SD and relationship satisfaction. What is more, most SD studies have examined specific subpopulations (e.g., separately by gender, individuals with a medical condition) [9, 28, 32] or specific SDs (e.g., lack of sexual interest) [28, 35], thereby failing to provide a comprehensive picture of SD correlates. Also, such studies did not compare clinical and nonclinical samples on SD correlates, limiting our understanding of possible specificities that could inform practice. Lastly, very few studies, to our knowledge, have explored whether rates of co-occurring SDs (i.e., comorbidity) are associated with increased sexual, relationship, and psychological distress [40]. Thus, the current scientific corpus presents partial or inconclusive findings on the psychosexual well-being of individuals with one or multiple SDs, especially of individuals consulting in sex therapy.

### Help-seeking for sexual dysfunctions and the characteristics of individuals who seek help

Although SDs are common, they remain under-reported and under-treated [3, 41]. In a large cross-national random population-based sample (*n* = 27,500), almost half of respondents (43% of men and 49% of women) had experienced at least one sexual difficulty in the last 12 months, though less than 20% had sought medical help for their problem [42]. Similarly, in a sample of women in the United States (*n* = 701), only half (53%) of those living with hypoactive sexual desire disorder sought professional help for their SD [22]. In another US female sample (*n* = 3,807; 18–75 years old), 40% (*n* = 1,519) of participants indicated not having sought medical help for SD-related complaints, although 54% reported that they would have wanted to do so [43].

While some research has documented individuals’ reasons for not seeking professional help (e.g., perceptions that SD is a normal part of aging or that it is a taboo subject) [3, 44–47] few studies have explored help-seeking behaviors, barriers to treatment, and the characteristics of individuals who seek services for SD. Some barriers to treatment appear to be related to screening processes and healthcare providers’ attitudes. In a sample of 300 gynecology patients (18–50 years old), while most (80%) reported wanting to be asked about their sexual health and functioning by their doctor, only one-third (36%) said their gynecologist had done so [48]. In a Swiss sample of gynecologists (*n* = 341), only 8% indicated routinely discussing sexual issues—including SD—with more than 80% of their patients [49].

Other potential barriers to sexual health services, such as structural (e.g., treatment costs, waiting times) and demographic factors (e.g., education, rurality), have been relatively underexplored. The few studies having examined such barriers have found age, education level [47], and gender [42] to be unrelated to seeking help for SD. By contrast, much research has been conducted on demographic (e.g., age, income, rurality) and structural barriers (e.g., waiting times, availability of services) to help-seeking behaviors and access for the treatment of non-sexual mental disorders (e.g., depression or anxiety disorders) [50–56]. Documenting help-seeking and its potential barriers and correlates is central to informing healthcare guidelines and policies [42]. It could allow for the identification of vulnerable subpopulations and foster the development of tailored strategies improving treatment access.

### The current study

To address the limitations of the current literature, this study’s aims were threefold: (1) to use disorder-level criteria to compare the prevalence of SD between a clinical sample of individuals consulting in sex therapy and a community-based sample; (2) to examine and compare sexual, relational, and psychological correlates of SD between samples; and (3) to examine help-seeking prevalence, barriers, and correlates in a community sample. We hypothesized that SD prevalence and levels of sexual, relational, and psychological distress would be higher among individuals consulting in sex therapy than in the community-based sample. Since the examination of help-seeking barriers and associated factors is descriptive and exploratory, no hypotheses were formulated.

## Materials and methods

The present study was approved by the Université du Québec à Montréal’s Institutional Ethics Review Board (approval number: 4829_e_2021; 1269_e_2017), and informed consent was obtained from each participant included in this study.

### Sampling

#### Community sample

A non-probabilistic adult sample of 2,154 Québécois (Canada) individuals was recruited via social media (i.e., *Facebook* and *Instagram*) from June to September 2021. Participants were invited to complete an anonymous online survey on sexual health and well-being in either French or English. Specifically, the community-based survey comprised ten sections assessing participants’ sexual difficulties (e.g., SD, problematic pornography consumption, sexualized drug use) and related issues (e.g., body shame, attachment insecurities, performance anxiety, sexual victimization), psychological and relational well-being, and barriers to treatment access. By clicking on the study link, participants were led to a consent form detailing the study’s nature and objectives, which they needed to review and sign electronically. After providing electronic consent, participants accessed the survey, hosted on *Qualtrics*. The survey took about 30 to 40 minutes to complete. Of the 2,154 participants who provided consent, 87.8% (*n* = 1,891) met the inclusion criteria, namely: (1) being at least 18 years old, (2) having sufficient knowledge of either French or English, and (3) completing at least 70% of the measures of interest. Individuals who did not meet these criteria were excluded from the present study. Participants were eligible to enter a draw to win one of 30 gift-cards with a value ranging from $25 to $200 CAD.

#### Clinical sample

Participants were recruited at the Université du Québec à Montréal’s sexology clinic (Québec, Canada) from December 2012 to May 2022. Patients (all adults) were invited by interns to complete an online self-reported survey hosted on *Qualtrics* during the evaluation phase of their treatment [19], which notably assessed levels of sexual functioning, sexual and relationship satisfaction, and psychological well-being. The informed consent procedure was the same as the one used in the community sample. Patients were informed that their refusal to participate in the study would not affect the access or quality of their care. The questionnaire was available in French and in English. Of the 1,093 participants who consented to participate, 88.1% (*n* = 963) met the inclusion criteria (i.e., identical to those used in the community sample).

### Measures

#### Sociodemographic characteristics

Sociodemographic data were collected on age, gender, sexual orientation, education, ethnicity, employment status, household income, relationship status, and religious practice and residential area (in the community sample only). The latter was assessed based on Statistics Canada’s [57] method of classification, which uses individuals’ postal codes.

#### Sexual dysfunctions

SD was assessed using the Arizona Sexual Experience Scale (ASEX) [58], which examines the experience of sexual difficulties throughout the sexual response cycle (e.g., sexual desire, erection/lubrication, orgasm) using a 6-point Likert scale ranging from 1 –*extremely easily/strong/satisfying* to 6 –*very difficult/weak/unsatisfying*. Lower scores represent greater levels of sexual functioning. Participants completed the version of the ASEX that corresponded to their genital sex rather than their gender (i.e., one’s personal sense of being male, female, non-binary, etc.), as some ASEX items are sex-specific (e.g., vaginal lubrication; penile erection). Two questions were added to the original ASEX to measure other sexual difficulties (i.e., pain during sex and premature ejaculation/orgasm). To reflect the diagnostic criteria used in the DSM-5 [11], investigated sexual difficulties had to be present for at least 6 months and respondents were invited to indicate their associated levels of distress (1 –*no distress*, to 6 –*extreme distress*). In the present study, five SDs were examined: 1) low sexual desire/arousal, 2) difficulties with lubrication/erection, 3) premature ejaculation/orgasm, 4) delayed or absent ejaculation/orgasm, and 5) pain during sex. The ASEX showed satisfactory internal consistency in the community (α = .82) and clinical samples (α = .75).

#### Sexual satisfaction

The Global Measure of Sexual Satisfaction (GMSEX) [59] was used to assess overall sexual satisfaction. Participants rated their sexuality on five 7-point bipolar scales ranging from: Bad-Good, Unpleasant-Pleasant, Negative-Positive, Unsatisfying-Satisfying, and Worthless-Valuable. Total scores ranged from 5 to 35, with higher scores indicating greater sexual satisfaction. The measure yielded satisfactory internal consistency in both the community (α = .91) and clinical samples (α = .89).

#### Relationship satisfaction

Relationship satisfaction was measured using the short 4-item Dyadic Adjustment Scale (DAS-4) [60]. Respondents rated their current relationship on conflict frequency on a 6-point scale ranging from 0 –*never* to 5 –*always*, and on levels of relationship happiness on a 7-point scale ranging from 0 –*extremely unhappy* to 6 –*perfect*. Total scores ranged from 0 to 21. Higher scores reflect greater relationship satisfaction. Internal consistency was satisfactory for the community (α = .81) and clinical samples (α = .75).

#### Psychological distress

The 6-item K-6 Distress Scale [61] was used in the community sample to measure symptoms of anxiety and depression. Participants rated the frequency of their symptoms using a 5-point scale ranging from 0 –*none of the time* to 4 –*all the time*. Total scores ranged from 0 to 24. Internal consistency was α = .87. The anxiety and depression subscales (8 items) of the Psychiatric Symptom Index [62] were used in the clinical sample. Participants rated the frequency of their symptoms on a 4-point scale ranging from 0 –*never* to 3 –*very frequently*. Total scores ranged from 0 to 24, with higher scores indicating greater psychological distress. Internal consistency was α = .89.

#### Help-seeking

Participants in the community sample were asked to indicate whether they sought professional help for their sexual difficulties, the types of help sought, and the potential barriers they encountered. Questions on sexual healthcare use and barriers to care were created based on previous studies [45, 47, 51, 56].

### Data processing and statistical analysis

To reflect the diagnostic criteria used in the DSM-5 [11], participants having selected at least 5 (very difficult/weak/unsatisfying) on a given ASEX sexual difficulty item with a score of at least 4 (moderate distress) for related distress were categorized as presenting that specific SD. Participants not meeting these criteria for a given SD were categorized as not having that specific SD. Total scores were used for the psychosexual variables (i.e., sexual and relationship satisfaction, and psychological distress).

Potential differences between samples on sociodemographic variables were explored using chi-square tests. Crude prevalence for each SD was calculated by dividing the total number of a given self-reported sexual problem by the total number of respondents. For each prevalence, 95% confidence intervals (CIs) were estimated using exact (Clopper-Pearson) confidence limits for a binomial proportion. Comparison analyses regarding SD prevalence and correlates were conducted using chi-square and independent samples *t*-tests. Associations between the number of reported SDs and all continuous psychosexual variables were tested using correlational analyses. Data on help-seeking and barriers to services are presented as frequencies and percentages. Missing data were omitted from analyses. Effect sizes were reported for each analysis (i.e., φ, Cohen’s *d*, and *r*) [63]. All statistical analyses were performed using SPSS, version 27, except for the 95% CIs for the crude prevalence, which were computed using the R package binGroup [64].

## Results

The demographic characteristics of the clinical (*n* = 963) and the community-based (*n* = 1,891) samples are summarized in Table 1. Samples slightly differed on age, sexual orientation, employment status, household income, and education level.

**Table 1:** Demographic characteristics of the community and clinical samples.

Variables	Community	Clinical	*p*-value (φ_c_)
	(*n* = 1891)	(*n* = 963)	
	% (*n*)	% (*n*)	
Age			
18 to 34	38.2 (702)	57.5 (553)	< .001 (.19)
35 to 49	36.7 (676)	27.4 (293)	
50 and over	25.1 (762)	15.1 (145)	
Gender			
Cis Women	54.7 (1035)	57.0 (549)	*n*.*s*.
Cis Men	41.2 (779)	40.0 (385)	
Other (e.g., non-binary, trans, etc.)	4.1 (77)	3.0 (29)	
Sexual orientation			
Heterosexual	75.5 (1427)	76.6 (734)	.006 (.07)
Gai/Lesbian	5.4 (102)	5.9 (57)	
Bisexual/Pansexual	14.4 (272)	11.1 (106)	
Asexual	1.3 (24)	.7 (7)	
Other (e.g., queer)	3.5 (66)	5.6 (54)	
Employment status			
Employed or self-employed	70.7 (1335)	62.5 (563)	< .001 (.18)
Student	10.8 (203)	24.0 (216)	
Unemployed/leave of absence	10.8 (203)	9.2 (83)	
Retired	6.1 (115)	4.0 (36)	
Other (e.g., volunteer, caregiver)	1.6 (31)	.3 (3)	
Household annual income (CAD)			
< $20,000	7.9 (123)	17.0 (125)	< .001 (.14)
$20,000-$59,999	33.4 (519)	34.1 (251)	
$60,000-$79,999	16.9 (263)	14.0 (103)	
$80,000-$99,999	16.0 (249)	13.3 (98)	
> $100,000	25.8 (402)	21.5 (158)	
Relationship status			
Single	35.5 (666)	31.8 (304)	*n*.*s*.
In a relationship	64.5 (1210)	68.2 (651)	
Education level			
Elementary school	1.5 (29)	2.0 (19)	.038 (.06)
High school	17.1 (323)	13.8 (132)	
Vocational school or college	39.9 (754)	37.7 (362)	
Undergraduate	30.9 (584)	35.4 (340)	
Graduate	10.6 (201)	11.1 (107)	

### Objective 1: Examine group differences in SD prevalence

SD prevalence for both samples is summarized in Table 2. Overall, individuals in the clinical sample were more likely than their community-based counterparts to report at least one SD and to report SD comorbidity. Moreover, all examined SDs were statistically more prevalent in the clinical sample. Effect sizes were small (φ = .05 to.17). Sample differences were particularly marked for low sexual desire/arousal (10.4% vs 18.9%, respectively) and delayed or absent orgasm/ejaculation (7.0% vs 18.6%, respectively). Symptom-level prevalence of at least one sexual impairment (i.e., *without* the distress criterion) was 30.1% in the community sample and 48.5% in the clinical sample, while disorder-level prevalence of at least one SD (i.e., *with* the distress criterion) was 19.6% in the community sample and 33.2% in the clinical sample.

**Table 2:** Group differences in SD prevalence and psychosexual well-being.

**Sexual dysfunctions**	**Community**		**Clinical**		
	% (95% CI)	Valid *n*^†^	% (95% CI)	Valid *n*^†^	φ (95% CI)
No SD	80.4 (78.6–82.2)	1521	66.8 (63.7–69.7)	643	.16*** (.13-.20)
1 SD	13.3 (11.8–14.9)	251	19.1 (16.7–21.7)	184	
2 SDs	3.9 (3.0–4.8)	73	9.1 (7.4–11.1)	88	
3 or more SDs	2.4 (1.8–3.2)	46	5.0 (3.7–6.6)	48	
Lack of sexual desire/arousal	10.4 (9.0–11.8)	195	18.9 (16.5–21.5)	182	.12*** (.08-.16)
Erectile/lubrication difficulties	6.1 (5.0–7.3)	105	8.5 (6.8–10.5)	78	.05* (.01-.08)
Premature ejaculation or orgasm	1.1 (0.7–1.7)	21	3.8 (2.6–5.5)	28	.09*** (.04-.13)
Delayed or absent ejaculation/orgasm	7.0 (5.9–8.3)	128	18.6 (15.8–21.6)	136	.17*** (.13-.22)
Pain during sex	6.2 (5.0–7.6)	93	9.5 (7.8–11.6)	91	.06** (.02-.10)
**Psychosexual variables**	**Community**		**Clinical**		
	*M* (*SD*)	Valid *n*^†^	*M* (*SD*)	Valid *n*^†^	*d* (95% CI)
Sexual satisfaction	24.3 (6.8)	1541	22.0 (7.1)	649	.35*** (.25-.44)
Relationship satisfaction	13.4 (5.0)	1541	13.3 (3.1)	649	.03 (.06-.12)
Psychological distress	7.6 (4.7)	1621	9.5 (5.7)	943	.36*** (.28-.44)

*Note*.

*p < .05,

**p < .01,

***p < .001.

^†^Valid *n* and % vary due to missing values (i.e., “prefer not to answer”).

### Objective 2: Examine group differences in sexual, relational, and psychological correlates of SD

Respondents in the clinical sample reported significantly lower levels of sexual satisfaction and greater degrees of psychological distress than the community sample (see Table 2). Effect sizes were moderate (*d* = .35 to.37). Estimated means for relationship satisfaction did not differ between samples. Psychosexual correlates of SD for both samples are summarized in Table 3. In the community sample, correlation analyses showed that the number of reported SDs was negatively related to sexual and relationship satisfaction, and positively related to psychological distress. In the clinical sample, the number of SDs was negatively related to sexual satisfaction only. Moreover, sexual and relationship satisfaction and psychological distress were significantly intercorrelated in both samples. Correlations were weak to moderate (*r* = .12 to.48).

**Table 3:** Psychosexual well-being correlates of sexual dysfunction by sample.

Variables	1	2	3	4	*M*	*SD*
***Community*** (*n =* 1,891)						
1. Number of SDs (0–4)^†^	—	-.43***	-.12***	.19***	0.3	0.7
2. Sexual satisfaction (5–35)		—	.48***	-.31***	24.3	6.8
3. Relationship satisfaction (0–21)			—	-.25***	13.4	5.0
4. Psychological distress (0–24)				—	7.6	4.7
***Clinical*** (*n =* 963)						
1. Number of SDs (0–4)^†^	—	-.36***	.03	.06	0.5	0.9
2. Sexual satisfaction (5–35)		—	.29***	-.21***	21.4	7.1
3. Relationship satisfaction (0–21)			—	-.28***	13.3	3.1
4. Psychological distress (0–24)				—	9.5	5.7

*Note*.

*p < .05,

**p < .01,

***p < .001.

^†^As participants could not report both delayed/absent and premature orgasm, the maximum number of SDs a participant could report was four (out of five).

### Objective 3: Examine help-seeking prevalence, barriers to services, and the characteristics of individuals seeking such services

One-fourth (26.6%) of participants in the community sample reported having sought professional services for their sexual difficulties (see Table 4). Of these participants, 60.4% said that they were able to receive such services. The professionals that were most sought out by participants were sex therapists (36.3%), general practitioners (18.9%), and psychologists (12.4%) (see Table 4). Most respondents who sought professional help experienced barriers to its access (58.7%), which were mainly high costs (25.9), long waiting lists (25.0%), and being unable to receive appropriate information (16.4%).

**Table 4:** Help-seeking behaviors and barriers to services among the community sample (*n =* 1,688).

Variables	% (*n*)
Have ever sought help	26.6 (449)
Were able to receive help	60.4 (271)
Professionals sought for help	
General practitioner	18.9 (49)
Medical specialist (urologist, gynecologist)	10.4 (271)
Psychologist	12.4 (32)
Sex therapist	36.3 (94)
Nurse	4.6 (12)
Physiotherapist	3.5 (9)
Social worker	3.5 (9)
Massage therapist	1.9 (5)
Other	8.5 (22)
Reported no barriers	41.3 (177)
Reported barriers	58.7 (252)
Too costly	25.9 (65)
Long waiting lists	25.0 (63)
Unable to receive appropriate information	16.4 (41)
Unable to get an appointment	9.9 (25)
Scheduling conflicts with work	5.6 (14)
Family responsibilities	3.2 (8)
Other	14.0 (36)

Regarding demographic characteristics, individuals who sought services were more likely to be cisgender men and trans and non-binary individuals, be non-heterosexual, adhere to a religious practice, and to be in a relationship (Table 5). No differences were found between people who sought services and those who had not with regards to age, employment status, household income, ethnicity, residential area, and education level. Respondents who had sought services reported significantly more SDs, lower levels of sexual and relationship satisfaction, and higher psychological distress compared to those who had not. Effect sizes for significant differences were low to moderate (φ = .06 to.11; *d* = .15 to.48).

**Table 5:** Demographic and psychosexual correlates of sexual health service-seeking in the community sample (*n =* 1,688).

	Sought services	Did not seek services	φ (95% CI)
Demographic variables	% (*n*)	% (*n*)	
Age			.03 (.01-.08)
18 to 34	36.6 (161)	40.1 (482)	
35 to 49	38.0 (167)	35.3 (424)	
50 and over	25.5 (112)	24.6 (296)	
Gender			.07* (.03-.13)
Cisgender women	50.1 (225)	57.9 (718)	
Cisgender men	44.3 (199)	38.2 (473)	
Other (e.g., non-binary, trans, etc.)	5.6 (25)	3.9 (48)	
Sexual orientation			.11*** (.07-.17)
Heterosexual	67.3 (302)	77.2 (957)	
Gay/Lesbian	6.7 (30)	4.7 (58)	
Bisexual/Pansexual	18.3 (82)	13.0 (161)	
Asexual	0.9 (4)	1.2 (15)	
Questioning/Other (e.g., queer)	6.9 (31)	3.9 (48)	
Employment status			.05 (.03-.11)
Employed or self-employed	70.4 (316)	69.6 (859)	
Unemployed	3.1 (14)	4.6 (57)	
Student	10.5 (47)	10.7 (132)	
Retired	6.5 (29)	6.1 (75)	
Sick leave	4.0 (18)	2.8 (34)	
Other (e.g., volunteering, caregiver)	5.6 (25)	6.3 (78)	
Household annual income (CAD)			.02 (.04-.12)
< $50,000	24.1 (108)	26.7 (329)	
> $50,000	65.6 (294)	62.8 (773)	
Missing data	10.3 (46)	10.5 (129)	
Ethnicity			.01 (-.04-.06)
White	94.4 (424)	94.0 (1165)	
Non-white	5.6 (25)	6.0 (74)	
Residential area			.04 (.01-.09)
Metropolitan area	67.8 (284)	66.8 (750)	
Other urban areas	9.3 (39)	11.8 (132)	
Rural	22.9 (96)	21.5 (241)	
Adheres to a religious practice			.06** (.01-.12)
Yes	19.4 (87)	14.2 (176)	
No	80.6 (362)	85.8 (1061)	
Relationship status			.06* (.02-.12)
Single	36.2 (162)	33.7 (416)	
In a relationship	60.3 (270)	64.5 (797)	
Other	3.6 (16)	1.8 (22)	
Education level			.04 (.02-.10)
Elementary/High school	16.3 (73)	18.6 (231)	
Vocational school or college	38.8 (174)	39.5 (489)	
Undergraduate	32.3 (145)	31.6 (392)	
Graduate	12.7 (57)	10.3 (127)	
	**Sought services**	**Did not seek services**	*p* (*d*)
**Psychosexual variables**	*M* (*SD*)	*M* (*SD*)	
Number of SDs	0.7 (1.1)	0.3 (.7)	.48*** (.59-.37)
Sexual satisfaction	22.8 (7.0)	24.8 (6.7)	.28*** (.17-.40)
Relationship satisfaction	12.8 (5.1)	13.5 (5.0)	.15** (.04-.26)
Psychological distress	8.20 (4.9)	7.4 (4.7)	-.16** (.27-.05)

*Note*.

*p < .05,

**p < .01,

***p < .001.

## Discussion

The present study compared the prevalence and correlates of SD between a clinical and a community sample, as well as examined help-seeking behaviors for SD, barriers to services, and individual characteristics associated with seeking professional services. That the clinical sample reported more SDs and were more likely to have used sex therapy services than the community sample is consistent with the finding that experiencing sexual difficulties is an important motive seeking sex therapy [18, 19]. More specifically, 9 to 19% of individuals in our clinical sample reported at least one SD, compared to 6 to 10% of individuals in our community sample. Rates of disorder-level SD in both samples are consistent with those found in other clinical [65] and community samples [15, 66–68], which suggests that they may be considered as reliable estimates of SD prevalence in community and clinical populations. The observed SDs prevalence confirmed that low sexual functioning represents an important public health issue, which may inform healthcare policies and guide clinicians (e.g., psychologists, general practitioners) during the screening process. Further, given the disparity between symptom- and disorder-level SD prevalence (i.e., a 3:2 ratio), the present findings underscore the importance of also using the distress criterion in SD research. Results also show that sex therapy clients report higher levels of psychological distress and lower levels of sexual satisfaction relative to individuals from the community, which is congruent with prior research [27, 69, 70]. Unexpectedly, both samples presented similar levels of relationship satisfaction. Observed DAS-4 means in both groups (i.e., community: 13.4, clinical: 13.3) fall between mean scores found in other sex therapy (<12) [60, 71] and non-clinical samples (> 15) [72–74], and are near threshold levels of those of clinically distressed couples (DAS-4 < 13) [60]. This finding suggests that relationship distress may not be specific to sex therapy clients. However, it is also possible that the COVID-19 pandemic, which occurred during the recruitment phase of the community sample, may have contributed to this sample’s lower levels of relational satisfaction compared to those found in previous population-based samples. Studies exploring the pandemic’s impact on relationships have indeed revealed increased sexual and relational distress and conflict between partners, as well as decreased relationship quality and frequency of intimate and sexual behaviors following the onset of the pandemic [75–78].

Consistent with other studies [32, 79], SD was associated with sexual and relational dissatisfaction and psychological distress, at least in the community sample. However, in the clinical sample, only sexual satisfaction was significantly related to lower numbers of reported SDs. This finding might be partially explained by the effect of confounding variables, such as financial hardship and adverse relational experiences (e.g., sexual assault, partner violence), both of which are particularly prevalent among sex therapy clients [19, 80] and have been found to be negatively associated with mental health and relational well-being [81, 82]. Since we found relational well-being to be more strongly related to sexual satisfaction than to sexual function in both samples, dyadic adjustment might be a less reliable indicator of sexual functioning than other relational factors such as (sexual) communication, sexual compatibility, partner’s sexual functioning or sexual skills, relational avoidance, romantic attachment, or levels of conflicts or coercion [8, 83–87]. Overall, in both samples, these findings confirm the particularly strong link between sexual functioning and sexual satisfaction [88], warranting their concurrent assessment in clinical and research settings.

The present study also found sex therapists to be the most consulted professionals—with one-third of respondents having sought these providers—followed by physicians (general practitioners and specialists combined; 29%) and psychologists (12%). While most respondents were able to access care for their SD, results suggest that nearly 6 out of 10 participants encountered barriers in accessing treatment. These findings highlight the importance of increased accessibility to affordable sexual health services, notably by addressing the identified structural barriers (e.g., long waiting times, cost of services). Moreover, individuals who sought services were mostly similar to those who did not in terms of sociodemographic characteristics. Only small differences were found in relation to gender, religious practice, and relationship status. This finding is consistent with that of previous studies [42, 47]. By contrast, respondents who had sought services reported significantly more SDs, lower sexual and relationship satisfaction, as well as higher psychological distress than individuals who had not sought services, suggesting that these factors may be more relevant to sexual health service-seeking than sociodemographic characteristics.

### Strengths and limitations

There has been little research comparing SD prevalence and their associated factors between clinical and community samples, especially using disorder-level DSM-5 criteria. Moreover, this study provides additional insights in the emerging research field of help-seeking for sexual difficulties and barriers to services. The study’s strengths also include its large, sexually- and gender-diverse samples.

Nonetheless, several limitations need to be considered. First, the study’s cross-sectional design precludes the drawing of any conclusions regarding the causality and directionality of the relationships between the examined variables. Second, the samples slightly differed on demographic characteristics (e.g., age, household income). Consequently, the findings should be interpreted with caution. Third, SD prevalence was estimated using self-reported questionnaires rather than official diagnostic records or clinical interviews. Thus, the results may be subject to social desirability and recall biases. Fourth, part of the recruitment occurred during the COVID-19 pandemic, which has impacted many individuals’ sex lives and relationships [76], thus affecting the findings’ generalizability to other contexts. Finally, structural barriers to treatment are intimately connected to the social and cultural contexts in which the study takes place (e.g., access to healthcare services and sexual education). Nevertheless, results suggest that when sex therapists are available, they tend to be favored over general practitioners and psychologists.

## Conclusion

The present research expanded the current SD literature by comparing clinical and community samples using a disorder-level definition of SD (i.e., persistent impaired sexual functioning causing significant distress), as previous studies have mainly examined the prevalence of SD symptoms rather than disorders as defined by the DSM-5 [2, 10, 16] in a single sample or in specific subpopulations (e.g., individuals suffering from a specific SD or medical condition) [89, 90]. Further, by examining demographic characteristics linked to help-seeking for SD as well as barriers to professional services, the current study contributes to the existing literature on help-seeking and treatment access, which has primarily focused on non-sexual mental health disorders [50, 53, 55]. Further research across multiple national and cultural contexts could shed additional light on barriers to sexual health services, as well as examine how the COVID-19 pandemic might have exacerbated SDs and influenced help-seeking behaviors. Future qualitative studies using in-depth interviews with individuals experiencing SDs (and eventually, with their partners) would allow for a more comprehensive understanding of their trajectory towards sexual health services and underlying barriers and motivations. Also, relationship between SD and medical conditions (e.g., endometriosis, infertility, cancer, urinary incontinence) [91–94] and other mental disorders (e.g., major depressive disorder, generalized anxiety disorder, substance use disorders) [95–97] should be investigated more thoroughly to provide a more comprehensive and multifaceted clinical picture of SD risk factors and comorbidities. Doing so could also provide further insight regarding the indirect sexual health service trajectories of individuals living with SDs, as many such individuals first seek help for a non-sexual condition (e.g., endometriosis, depression, etc.) that can negatively impact sexual function. Finally, additional research could examine whether the nature of the motive of consultation (e.g., sexual or mental health disorder) influences help-seeking behaviors, as well as explore other potential associated factors, such as stigma and sex education.
